# Mechanism of Nucleic Acid Sensing in Retinal Pigment Epithelium (RPE): RIG-I Mediates Type I Interferon Response in Human RPE

**DOI:** 10.1155/2021/9975628

**Published:** 2021-06-18

**Authors:** Joshua Schustak, Michael Twarog, Xiaoqiu Wu, Henry Y. Wu, Qian Huang, Yi Bao

**Affiliations:** The Department of Ophthalmology, Novartis Institutes for BioMedical Research, 22 Windsor Street, Cambridge, MA, USA

## Abstract

Age-related macular degeneration (AMD), a degenerative disease of the outer retina, is the leading cause of blindness among the elderly. A hallmark of geographic atrophy (GA), an advanced type of nonneovascular AMD (dry AMD), is photoreceptor and retinal pigment epithelium (RPE) cell death. Currently, there are no FDA-approved therapies for GA due to a lack of understanding of the disease-causing mechanisms. Increasing evidence suggests that chronic inflammation plays a predominant role in the pathogenesis of dry AMD. Dead or stressed cells release danger signals and inflammatory factors, which causes further damage to neighboring cells. It has been reported that type I interferon (IFN) response is activated in RPE cells in patients with AMD. However, how RPE cells sense stress to initiate IFN response and cause further damage to the retina are still unknown. Although it has been reported that RPE can respond to extracellularly added dsRNA, it is unknown whether and how RPE detects and senses internally generated or internalized nucleic acids. Here, we elucidated the molecular mechanism by which RPE cells sense intracellular nucleic acids. Our data demonstrate that RPE cells can respond to intracellular RNA and induce type I IFN responses via the RIG-I (DExD/H-box helicase 58, DDX58) RNA helicase. In contrast, we showed that RPE cells were unable to directly sense and respond to DNA through the cGAS-STING pathway. We demonstrated that this was due to the absence of the cyclic GMP-AMP synthase (cGAS) DNA sensor in these cells. The activation of IFN response via RIG-I induced expression of cell death effectors and caused barrier function loss in RPE cells. These data suggested that RPE-intrinsic pathways of nucleic acid sensing are biased toward RNA sensing.

## 1. Introduction

Age-related macular degeneration (AMD) is the most common cause of blindness among the elderly in the US and worldwide [1]. Although antiangiogenic drugs are currently in use for wet AMD, no Food and Drug Administration- (FDA-) approved treatments for intermediate AMD or geographic atrophy (GA) exist to date. A hallmark of AMD and GA is photoreceptor and retinal pigment epithelium (RPE) cell death. RPE, consisting of critical retinal cells and involved in AMD, is a unique epithelial tissue that interacts with photoreceptors on its apical side and with Bruch's membrane and the choriocapillaris on its basal side, making it essential for the maintenance of vision. Aging and other accumulated genetic and environmental risk factors might lead to RPE dysfunction, eventually resulting in RPE degeneration, and ultimately cell death, a major pathology of AMD. However, the cellular mechanisms causing degeneration of RPE are poorly understood.

Increasing evidence have indicated that chronic inflammatory events mediated by key pathways (e.g., complement, Toll-like receptor (TLR), NF-*κ*B, inflammasome, and interferon (IFN) response) play a central role in the pathogenesis and development of AMD [2, 3]. However, the molecular nature of these inflammatory mechanisms remains unclear. The immune status of the chorioretinal interface is known to be mainly regulated by RPE, with inflammation potentially having both physiological and pathological roles in RPE maintenance. Recent studies have suggested that type I IFN response is activated in RPE in patients with AMD. It was shown that both the mRNA and protein levels of IFN-*β* were increased in the RPE of patients with GA compared with age-matched controls [4]. In addition, it was reported that type I IFN pathway played an important role in animal models of retinal degeneration, most notably in A/J mice, as well as following the Alu-RNA subretinal delivery [4, 5]. However, the mechanisms by which RPE cells sense nucleic acids and induce downstream type I IFN response in the retina have not been fully addressed.

The type I family of IFNs is known to be responsible for inducing an antiviral response, playing a major role in restricting virus-infected cells. In addition to responding to foreign viral infections, they have also been implicated in sensing self-nucleic acids in the form of danger-associated molecular patterns (DAMPs). In particular, DAMPs have been shown to be released from dying or stressed cells and detected by pattern recognition receptors (PRRs) of the innate immune system. DAMPs induce inflammation and further damage to other cells. In mammalian cells, recognition of nucleic acids is known to involve multiple sensors. Specifically, members of the TLR family have been reported to sense RNA through TLR3, TLR7, and TLR8 and DNA via TLR9. In cytosol, RNA sensing is known to mainly occur through RIG-I-like receptors (RLRs), a group of RNA-specific PRRs. Intracellular DNA sensing, however, has been shown to mainly occur through the cGAS/STING axis, which detects cytosolic double-stranded DNA (dsDNA) [6, 7]. Once activated, these nucleic acid sensors trigger multiple signaling cascades and produce type I IFNs and proinflammatory cytokines via the activation of the interferon regulatory factor (IRF) and NF-*κ*B family of transcription factors [8, 9]. It was reported that treatment of poly(I:C) in culture medium could induce IRF3-mediated IFN-*β* and NF-*κ*B-mediated cytokine release through TLR3 and further generate interferon stimulate genes (ISGs) in both ARPE-19 and primary human RPE cells [10, 11]. However, the mechanism by which this system of nucleic acid sensing works in RPE, as well as which sensors are responsible for internally generated or internalized nucleic acids and activating the type I IFN pathway in the retina, remains unknown.

In this study, we confirmed that RPE could both produce and respond to interferon-beta (IFN-*β*), leading to downstream signaling. Surprisingly, whereas RPE cells were demonstrated to directly respond to intracellular RNA, they did not respond directly to DNA due to lack of expression of the cyclic GMP-AMP synthase (cGAS) DNA sensor. We further identified the RNA sensor RIG-I (also known as DExD/H-box helicase 58, DDX58) as a key sensor for initiating the type I IFN response in RPE.

## 2. Materials and Methods

### 2.1. Human samples

Postmortem human eyes, including patients with geographic atrophy (GA) and non-AMD controls, were procured from the Lions Eye Institute for Transplant & Research (LEITR; Tampa FL) with consent of donors or donors' next of kin and in accordance with the law of US and Florida, the Declaration of Helsinki and FDA regulations, and Novartis guidelines of using human tissues in research. All study tissues were preserved within 6 hours postmortem or less. The donor information used for IHC and RNAscope is listed in Supplementary Table 1.

### 2.2. Immunohistochemistry (IHC) and RNAscope

For IHC, the eyes were fixed in Modified Davidson's Fixative (MDF, H0290-500ML, Sigma-Aldrich, St. Louis, MO) for 48 hours, followed with 70% alcohol for an additional 48 hours all at room temperature. The eyes were embedded in paraffin wax, serially sectioned at a thickness of 5 *μ*m through the optic nerve, and processed for antibody staining using Leica Bond RX according to manufacturer's instructions. ×20 and ×40 images were taken by the Aperio AT2 scanner. The specificity of RIG-I (LsBIO LS-C344928, Lifespan Biosciences, Seattle, WA) antibody was validated using A549-Dual and A549-Dual KO-RIG-I cells (Supplementary Figure 1A-B) (InvivoGen, San Diego, CA). IgG antibody was used as a negative control (Supplementary Figure 1C).

For RNAscope, in situ hybridization on MDF-fixed paraffin-embedded retinal samples was performed using the RNAscope® 2.5 LS-Reagent Kit-RED (Advanced Cell Diagnostics, Hayward, CA) with RIG-I (NM_550268) specific probe according to the manufacturer's instructions. Probes for PPIB and DapB (Advanced Cell Diagnostics) were used as positive (data not shown) and negative controls (Supplementary Figure 1D), respectively.

### 2.3. Image analysis for IHC and RNAscope

IHC slides were scanned with Aperio at ×40 magnification, and scans were analyzed with the HALO image analysis platform (Indica labs, Albuquerque, New Mexico, USA). Analysis setting was Area quantification v1.0, and the area to be analyzed was assigned with circular annotation layers placed over the ONL and RPE layer in macula region.

RNAscope slides were scanned with Aperio at ×40 magnification, and scans were analyzed with the HALO image analysis platform (Indica Labs, Albuquerque, New Mexico, USA) using the Chromogenic RNA ISH Module. This allowed for thresholding and detection of positive probe staining on a single-cell basis to quantify average number of probe copies per cell. The area to be analyzed was assigned with circular annotation layers placed over all the layers in macula region.

### 2.4. Cell cultures

ARPE-19 was purchased from ATCC (Manassas, VA; CRL-2302). ARPE-19 was cultured in DMEM/F12 (Gibco Life Technologies, Carlsbad, CA) supplemented with 10% heat-inactivated fetal calf serum (FBS, Sigma-Aldrich) and 1% penicillin/streptomycin (Gibco BRL, Grand Island, NY) at 37°C and 5% CO_2_. Cells were cultured at least 3 weeks postseeding to form mature monolayers unless otherwise specified. Cell morphology was monitored daily, and mycoplasma was measured by MycoAlert Mycoplasma Detection Kits (Lonza, Basel, Switzerland) every other month to avoid contaminations. iCell-RPE, an iPS-derived RPE cell line, was purchased from FUJIFILM Cellular Dynamics (Madison, WI). Cells were cultured in Lonza RtEGM medium (Lonza) at 37°C and 5% CO_2_. iPS-RPE cells were cultured on tissue culture treated plates, or on Falcon transmembrane inserts for polarization (Corning, Corning, NY; 353095). Cells were cultured at least 3 weeks postseeding to form mature monolayers. Maturation of cells was confirmed by pigmentations, immunofluorescence staining for ZO-1 (Thermo Fisher Scientific, Waltham, MA #33-9111) and BEST1 (Novus, #NB300-164), and transepithelial resistance (Supplementary Figure 2).

THP1-Dual, THP-1-Dual KO-cGAS, A549-Dual, and A549-Dual KO-RIG-I cells were purchased from InvivoGen, and cells were prepared and cultured following the manufacturer's protocols. KO cell lines were validated by western blotting.

### 2.5. Generation of ARPE-19 RIG-I KO cell line

ARPE-19 cells stably expressing the Cas9 gene (ARPE19-Cas9) were generated by lentiviral transduction of ARPE-19 cells. Virus was harvested from supernatants of HEK-293T cells (ATCC) cotransfected with the in-house lentiviral vector pNGx_LV_c010 [12] and Lentiviral Packaging Plasmid Mix (Cellecta, Mountain View, CA; #CPCP-K2A) using TransIT-293 Transfection Reagent (MirusBio, Madison, WI). ARPE19-Cas9 cells were selected with 125 *μ*g/mL HygromycinB (Invitrogen, Carlsbad, CA), and Cas9 expression was verified by western blotting using Anti-FLAG primary antibody (Sigma-Aldrich #F1804, Supplementary Figure 3A-B). ARPE-19 RIG-I KO cells were generated by sgRNA expression in ARPE-19-Cas9 cells by lentiviral infection using the in-house lentiviral vector pNGx_LV_g003 [12]. The sgRNA sequence targeting DDX58/RIG-I is ACTCACCCTCCCTAAACCAG. Control sgRNA sequences were as follows: Ctrl1, GTAGCGAACGTGTCCGGCGT; Ctrl2, GACCGGAACGATCTCGCGTA; Ctrl3, GGCAGTCGTTCGGTTGATAT; and Ctrl4, GCTTGAGCACATACGCGAAT. Knockout was verified by western blot analysis (Supplementary Figure 3C).

### 2.6. Reagents

IFN-*α*, IFN-*β*, IFN-*γ*, and IFN-*λ*1/2/3 were purchased from R&D Systems (Minneapolis, MN). Anti-IFN-*β* antibody (InvivoGen) was used to neutralize IFN-*β*. dsDNA-EC, ISD (interferon stimulatory DNA), polydA:dT, poly(I:C)-LMW, and 3p-hpRNA were purchased from InvivoGen. Total nucleic acids (NA) were isolated from ARPE-19 using Nucleic Acid Isolation Kits (Thermo Fisher Scientific) according to the manufacturer's instructions. Transfection efficiency of each inducer (~80%) was confirmed using imaging of fluorescence labeled nucleic acids in ARPE-19 and iPS-RPE cells.

### 2.7. Western blot analysis and enzyme-linked immunosorbent assay (ELISA)

Culture supernatants were harvested, and whole-cell lysates were extracted using RIPA cell lysis buffer (Cell Signaling Technology, Danvers, MA) for western blotting or cell lysis buffer (CST#9803) for ELISA, supplemented with protease and phosphatase inhibitors (Thermo Fisher Scientific) according to the manufacturer's protocol. All samples were stored at −80°C before use.

For western blotting, samples (30 *μ*g of protein) were dissolved in sample buffer (Invitrogen) and boiled for 5 min at 100°C. The sample was then separated by Criterion™ TGX™ precast gel electrophoresis and electrotransferred onto nitrocellulose membranes (Bio-Rad, Hercules, CA). The MagicMark™ XP Western Protein Standard (Invitrogen) was used as a molecular weight indicator. The membrane was blocked for 1 h using 5% milk in Tris-buffered saline and Tween 20 (TBST). Following three washes with TBST, the membrane was incubated overnight with primary antibodies purchased from Cell Signaling Technology, including cGAS (CST#15102), IRF3 (CST#11904), RIG-I (CST#3743), Histone H3 (CST#4499), *β*-actin (CST#5125), and GAPDH (CST#8884), at 1 : 1000 dilution. After three washes with TBST, the membrane was incubated with a secondary antibody (anti-rabbit IgG-HRP-linked antibody, at 1 : 1000 dilution; Cell Signaling Technology) for 1 h. The immunoreactive proteins were detected using the SuperSignal™ West Femto Maximum sensitivity substrate (Thermo Fisher Scientific) and imaged on FluorChem M (ProteinSimple, San Jose, CA).

For ELISA, lysates or supernatants were measured in 384-well plates using antibody pairs for ISG15 (R&D #AF4845, #A-830), cGAS (Cayman #23853, CST#66546), IFN-*α*1 (Abcam #ab215408), IFN-*β* (R&D #DY814), IFN-*γ* (R&D #DY285B), IFN-*λ*1/3 (R&D #DY1598B), or Human IL-6 DuoSet for IL-6 (R&D #DY206). For some experiments, assays were run using Meso Scale Discovery plates (Meso Scale Diagnostics, Rockville, MD), and IFN-*β* secretion in supernatant was analyzed using the Human IFN-*β* Tissue Culture Kit (MSD #K151ADB). Protein levels were measured according to manufacturer instructions.

### 2.8. RNA interference

RNA interference against key nodes of the type I IFN pathway was performed using Qiagen (Hilden, Germany) or Dharmacon (Lafayette, CO) small interfering RNAs (siRNAs). The AllStars and Negative Control siRNAs were purchased from Qiagen and used as negative controls. Transfection of siRNAs in ARPE-19 cells was carried out using Dharmafect 4 (Dharmacon) according to the instructions of the manufacturer. Cells were transfected with 1 pmol/96-well culture dish for IFN-*β* release measurement by the IFN-*β* Human Tissue Culture Kit (Mesoscale Discovery, Rockville, MD). At least three different siRNAs were used for targeting each key nodes of the pathway. The knockdown efficiency was validated using real-time reverse transcription- (RT-) PCR.

### 2.9. Real-Time RT-PCR

Messenger RNAs were isolated from ARPE-19 cells treated with indicated siRNAs using the TurboCapture 96 mRNA Kit (Qiagen), and RNA was reverse transcribed using the High-Capacity cDNA Reverse Transcription Kit (Applied Biosystems, Foster City, CA). Real-time PCR was performed using FAM-labeled TaqMan probes targeting genes of interest and a VIC-labeled TaqMan probe targeting *β*-actin as control (Applied Biosystems). Reactions were run with TaqMan Universal PCR Master Mix on the ViiA7 system (Applied Biosystems) according to the instructions of the manufacturer.

### 2.10. RPE cell death

RPE cell death was measured using CytoTox-Glo (Promega, Madison, WI) or propidium iodide (PI, Thermo Fisher Scientific) staining and real-time monitoring using IncuCyte (Essen BioScience, Ann Arbor, MI). For CytoTox-Glo, cells were treated with indicated stimuli for 2, 6, 24, and 72 h, and then supernatants were collected and measured by CytoTox-Glo according to the instructions of the manufacturer. To monitor PI staining in real time, cells were treated with indicated stimuli and imaged in IncuCyte for bright field and red channel every 3 h according to the instructions of the manufacturer. Images were analyzed using the IncuCyte software.

### 2.11. Transepithelial resistance (TER)

Barrier function of RPE cells was assessed by monitoring TER every 15 minutes by means of a cellZscope 2 (NanoAnalytics GmbH, Münster, Germany). The resistance values for individual monolayers at specific times (*Ω*cm^2^) were determined, subtracted for background resistance produced by the blank filter and culture medium (as 0%), and normalized to baseline resistance prior to stimulation (as 100%).

### 2.12. Data Analysis

Protein levels measured by ELISA were presented as absolute amount, and gene levels by qPCR were normalized by *β*-actin. Three independent experiments with triplicates within each experiment were performed, and values were presented as mean ± SD. Two groups were compared using Student's *t*-test. Multiple comparisons were made using analysis of variance followed by the post hoc *Newman–Keuls* test. Differences were considered significant at *p* < 0.05.

## 3. Results

### 3.1. Retinal pigment epithelium cells both produced and responded to type I interferon

Previous reports have shown that the type I IFN pathway is activated in patients with GA and enriched in RPE [4]. We thus decided to focus on the mechanisms through which such a response might be potentiated in RPE. We used ARPE-19, a spontaneously arising RPE cell line, and induced pluripotent stem- (iPS-) derived RPE cells from multiple donors in our in vitro assays. Although ARPE-19 is a widely used human-derived RPE-like cell line, it has been reported to lack some features of mature RPE, such as pigmentation and expression of *RPE65*, while retaining some RPE features such as morphology, polarization, and phagocytosis [13]. Due to these limitations, we also utilized iPS-derived RPE cells to further support our interrogations. Prior to performing experiments, we confirmed the characteristics of mature RPE in iPS-RPE cells (Supplementary Figure 3), including barrier function, pigmentation, morphology, and expression of mature RPE markers (bestrophin 1 (*BEST1*), etc.).

**Figure 1:**
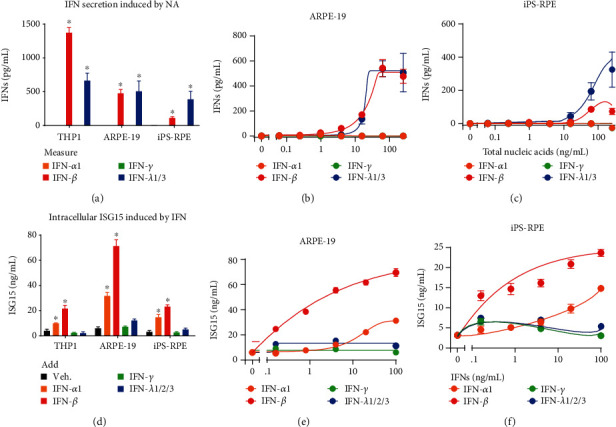
RPE cells respond to nucleic acids to secrete IFNs and also respond to secreted IFNs to generate ISGs. (a–c) Nucleic acid challenge-induced production of IFN-*β* and IFN-*λ* in RPE. Total nucleic acids (NA) were isolated from ARPE-19 cells and transfected into indicated cell types at 0.25 *μ*g/mL. Secreted IFN proteins were measured using ELISA 24 h after stimulation; only IFN-*β* and IFN-*λ* were predominantly produced in RPE cells. Examples of dose-response curves in ARPE-19 (b) and iPS-RPE (c) cells. (d–f) RPE cells respond to IFN-*α* and IFN-*β* to initiate downstream signaling and generate ISGs. Several key types of IFNs (100 ng/mL each) were added to RPE cells, with RPE cells primarily responding to IFN-*α* and IFN-*β* by inducing intracellular ISG15 measured by ELISA 24 h after stimulation. Examples of dose-response curves in ARPE-19 (e) and iPS-RPE (f) cells. Each data point represents biological replicates (*n* = 3), and is indicated as mean ± SD. ^∗^*p* < 0.05 compared with relative vehicle control groups.

There are 3 major types of IFN family members: type I (IFN-*α* and IFN-*β*), type II (IFN-*γ*), and type III (IFN-*λ*). To determine exactly which types of IFN are stimulated by exposure to nucleic acids in RPE, we measured the secretion of IFNs. We used a total cell nucleic acid (NA) fraction, including both DNA and RNA, to stimulate both ARPE-19 and iPS-RPE cells. We then detected the secretion of both IFN-*β* and IFN-*λ* in NA-transfected RPE cells (Figures 1(a)–1(c)) using ELISA, hence confirming that RPE could sense nucleic acids to generate IFNs. In addition, to determine whether IFNs participate in an autoregulatory feedback loop within RPE, we treated cells with recombinant IFNs and measured the induction of intracellular ISG15, one of the most strongly and rapidly induced interferon-stimulated genes (ISGs) [14–16], using ELISA. Upon treatment with different IFN types, our results indicated that RPE cells responded to type I IFN with downstream activation of ISG15 (Figures 1(d)–1(f)). Taken together, these data showed that RPE cells have the necessary components to both produce IFN, namely IFN-*β* and IFN-*λ*, and respond to IFN, primarily IFN-*β*, via the induction of downstream IFN signaling.

### 3.2. Identification of RIG-I as a major sensor of intracellular RNA in retinal pigment epithelium

**Figure 2:**
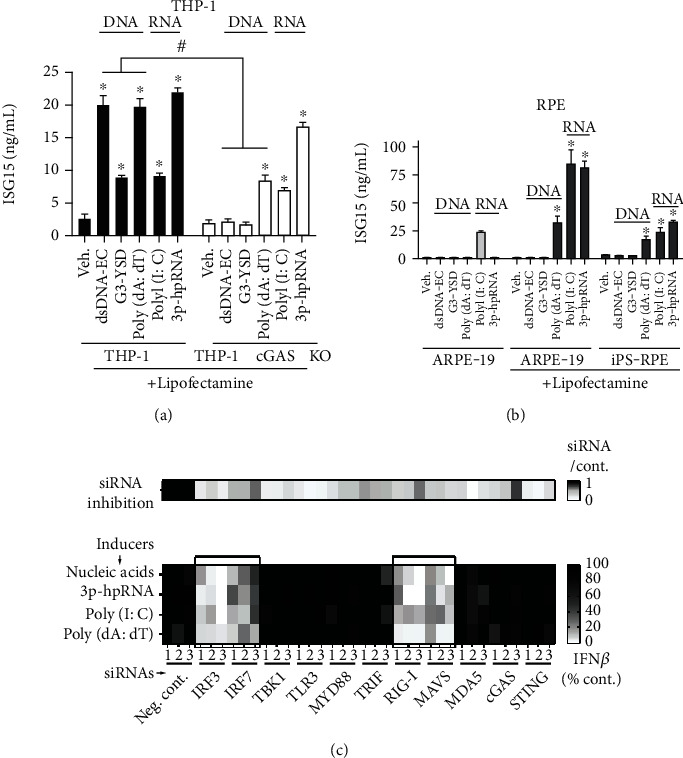
Identification of RIG-I as the key sensor in type I IFN response in RPE. (a, b) RNA but not DNA could be sensed by RPE to induce type I IFN response, suggesting the lack of DNA sensors in unstimulated RPE. THP-1 and THP-1 cGAS KO cells were used as controls (a). Cells were treated with indicated inducers at 0.25 *μ*g/mL, with or without the Lipofectamine transfection reagent, and intracellular ISG15 was measured by ELISA 24 h after stimulation. Each bar represents biological replicates (*n* = 3) and is indicated as mean ± SD. Note that ARPE-19 without transfection did not respond to most tested inducers except for poly(I:C). ^∗^*p* < 0.05 compared with corresponded vehicle-treated groups. ^#^*p* < 0.05 compared with same induces in parental cells. (c) Type I IFN response was induced via the RIG-I–MAVS–IRF3 axis in ARPE-19 cells. ARPE-19 cells were cultured 10 d after reaching confluence for screening purposes. Different key nodes for the activation of the IFN pathway were knocked down using siRNAs (at least 3 different siRNAs for each gene). Cells were then treated with indicated nucleic acids, and the release of secreted IFN-*β* was measured by ELISA 24 h after stimulation. Similar results were observed in 3 independent experiments with different inducers (indicated in different lines) or different siRNAs (shown as different column); a single representative experiment is shown as an example. A heatmap was used to better visualize the percentage (%) change in the results. The efficiency of the mRNA knockdown was validated by qPCR and shown at the top in the form of heatmap. About ~70% inhibition was observed in most tested siRNAs.

Upon showing that RPE cells could both produce and respond to IFN, we next sought to determine the sensors responsible for recognition of nucleic acids, including both DNA and RNA. First, we used THP-1 cells to validate the quality of nucleic acid inducers (either DNA or RNA) used in RPE cells (Figure 2(a)). To this end, we challenged RPE cells with DNA or RNA, with or without transfection reagent, in order to capture both cell surface and internal NA sensors [4, 11, 17–19]. As shown in Figure 2(b), in the absence of transfection reagent, ARPE-19 cells did not respond to any inducers tested except for poly(I:C), which was consistent with previous reports that RPE responded to extracellular poly(I:C) through TLR3, a reported RNA sensor [10, 11, 19]. Next, we wanted to characterize the intracellular NA sensors in RPE cells. Specifically, we aimed to determine whether RPE cells could detect intracellular DNA, RNA, or both. To our surprise, we observed that in the presence of transfection reagent, both ARPE-19 and iPS-RPE cells responded to RNA but not DNA except for poly(dA:dT), a repetitive synthetic double-stranded DNA sequence (Figure 2(b)). This response was greater than that to nucleic acids applied without transfection reagent, which is consistent with a similar observation in endothelial cells [20]. Interestingly, as shown in Figure 2(a), poly(dA:dT)-induced IFN response was also demonstrated to only be partly reduced in THP-1-Dual KO-cGAS cells, suggesting that poly(dA:dT) might be recognized and initiate downstream signaling indirectly through a nonclassic DNA sensing system. This agreed with previous reports that poly(dA:dT) could indirectly activate IFN pathways through an RNA polymerase III-transcribed RNA intermediary [17, 21].

In order to pinpoint specific intracellular nucleic acid sensors in RPE, we performed an in vitro siRNA knockdown screen targeting key nodes of the IFN pathway in ARPE-19 cells. To this end, we tested different nucleic acids, including commercially available DNA/RNA mimics or isolated total nucleic acid content from cells as inducers, and evaluated the consequent secretion of IFN-*β*. We used a knockdown of the interferon regulatory factor 3 (IRF3) transcription factor, one of the master regulators of IFN [9, 22], as our positive control. We accordingly noted that IRF3 knockdown significantly inhibited the induction of the secretion of IFN-*β* (Figure 2(c)). Interestingly, we found that the most pronounced reduction in the secretion of IFN-*β* was observed in both RIG-I and MAVS knockdown groups (Figure 2(c)), regardless of whether RPE was stimulated with RNA or DNA or a RNA/DNA mixture. These results indicated that the RIG-I-mediated signaling pathway is responsible for sensing intracellular nucleic acids in RPE cells.

**Figure 3:**
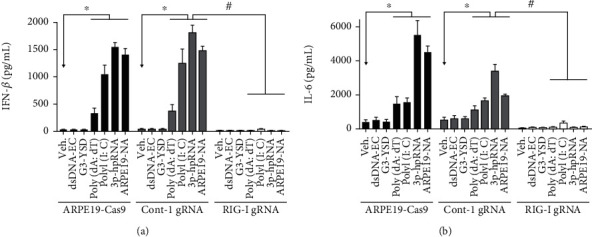
ARPE-19 cannot sense nucleic acids in RIG-I-deficient cells. The ARPE-19 RIG-I KO cell line was generated and validated using an ARPE-19-Cas9 line (see Supplementary Figure 3). Cells were stimulated with indicated inducers at 0.25 *μ*g/mL, and the release of IFN-*β* or IL-6 was measured by ELISA 24 h after stimulation. (a) The IFN response was abolished in RIG-I KO ARPE-19 cells. Similar to ARPE-19, Cas9-expressing ARPE-19 cells respond to RNA but not DNA. Interestingly, ARPE-19 RIG-I KO cells did not respond to any tested nucleic acids, further validating RIG-I as the major sensor in RPE cells. (b) The nucleic acid-induced release of IL-6 was also reduced in ARPE-19 RIG-I KO cells. Note that unlike other stimuli, the poly(I:C)-induced release of IL-6 was only partly reduced in RIG-I KO cells, suggesting the potential involvement of other sensors. ^∗^*p* < 0.05 compared with corresponded vehicle-treated groups. ^#^*p* < 0.05 compared with same inducers in control gRNA-transduced cells.

We performed further validation of the importance of RIG-I in the recognition of intracellular nucleic acids using a CRISPR/Cas9 KO in ARPE-19 cells. We generated an ARPE-19 cell line stably expressing Cas9 and control or RIG-I gRNA-transduced cells and validated them by western blotting (Supplementary Figure 3). Compared with parental cells, control gRNA-transduced cells were demonstrated to have a similar profile of nucleic acid sensing. However, the secretion of IFN-*β* in RIG-I gRNA-transduced cells was shown to be completely abrogated (Figure 3(a)), confirming the role of RIG-I as a key sensor for type I IFN response in RPE cells in vitro.

In addition to IFN, RIG-I sensing of nucleic acids has also been shown to activate NF-*κ*B-mediated cytokine release, including interleukin 6 (IL-6). It was reported that RIG-I could directly regulate the expression of NF-*κ*B [23] or indirectly regulate the activation of NF-*κ*B through mitochondrial antiviral signaling protein (MAVS) and TANK-binding kinase 1 (TBK1) [24, 25]. Therefore, we used the secretion of IL-6 as a readout to evaluate the nucleic acid-induced RIG-I-mediated activation of NF-*κ*B in RPE cells. We found that most of the secretion of IL-6 was abolished in RIG-I KO cells, indicating that the NF-*κ*B-mediated cytokine release was RIG-I-dependent. Interestingly, in contrast to the IFN response, poly(I:C)-induced IL-6 release was shown to be only partially reduced in ARPE-19-RIG-I KO (Figure 3(b)), suggesting another RNA sensor, that is, TLR3, might be responsible for this poly(I:C)-induced cytokine release in RPE cells, in agreement with previous reports [11]. Furthermore, we noted that the poly(dA:dT)-induced response in ARPE-19 was abolished in RIG-I siRNA-treated or ARPE-19-RIG-KO cells, as shown in Figures 2(c) and 3(a), respectively, further confirming the RIG-I-dependent response of ARPE-19 cells to poly(dA:dT).

**Figure 4:**
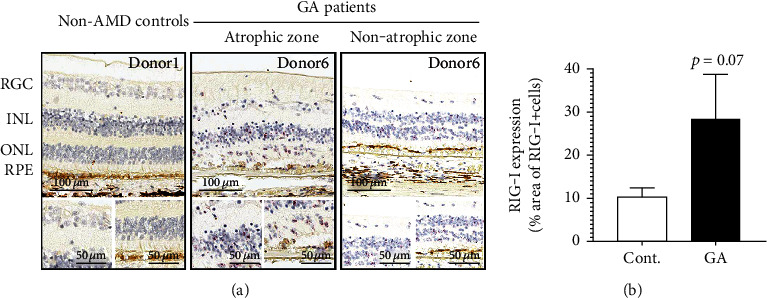
RIG-I mRNA is elevated in patients with GA. (a) The elevation in the levels of RIG-I mRNA in patients with GA relative to non-AMD controls was observed using RNAscope. Low levels of RIG-I signal (red dots) were detected in non-AMD control patients, whereas elevated signals were detected in RGC, ONL, INL, and RPE layers of patients with GA. Scale bars are indicated. *n* = 3/group, patient information is listed in Supplementary Table 1. DAPB was used as negative control (see Supplementary Figure 1D). In addition, the RIG-I signal in the macular region was analyzed by HALO, with the % area of RIG-I positive signals being shown in (b).

**Figure 5:**
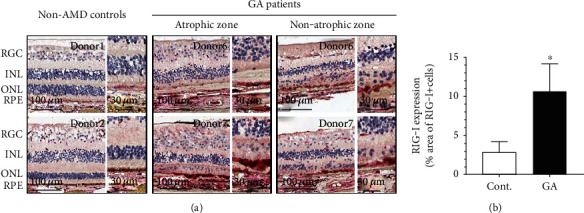
The RIG-I protein is expressed in human RPE, and elevated in patients with GA. (a) The expression of the RIG-I protein in non-AMD control and patients with GA was measured using IHC. Note that, unlike mRNA, RIG-I protein (red) was mainly enriched in RPE and choroid. The IgG control was used as negative control (see Supplementary Figure 1C). Examples of region selection for IHC and RNAscope from donor 6 (patient with GA) are shown in Supplementary Figure 4. In addition, the RIG-I signal in the macular region was analyzed by HALO, with the % area of RIG-I positive signals being shown in (b). Representative pictures of RIG-I IHC for each donor are shown in Supplementary Figure 5. Scale bars are indicated. *n* = 5/group, and patient information is listed in Supplementary Table 1. ^∗^*p* < 0.05 compared with non-AMD control patients.

Our findings suggested that RIG-I is the key NA sensor in RPE cells. To determine whether these in vitro findings were relevant to AMD, we first sought to determine whether the expression of RIG-I was increased in samples from donors with AMD. We used RNAscope and immunohistochemistry (IHC) to analyze retinal sections from donors with AMD. We observed that the mRNA expression of RIG-I was low but detectable in control human donors, whereas it significantly upregulated in the retina of patients with GA, notably in retinal ganglion cells (RGCs), outer nuclear layer (ONL), inner nuclear layer (INL), and RPE layers (Figure 4). This was consistent with the role of RIG-I as a well-known mammalian ISG [26]. In order to further determine the expression of the RIG-I protein in human donors, we validated a RIG-I antibody for IHC using the A549 RIG-I KO cell line (Supplementary Figure 1A-B). We consistently observed that the levels of the RIG-I protein in RPE cells were increased in all 5 patients with GA compared with control donor samples (Supplementary Figure 5 and Figure 5), supporting the key role of RIG-I in mediating type I IFN response in RPE cells. Surprisingly, we found that in contrast to the transcript, the RIG-I protein was almost exclusively observed in RPE and choroid layers in both patients with GA and non-AMD controls (Figures 4(a) and 5(a)), suggesting that posttranscriptional mechanisms might be involved in regulating the expression of RIG-I in non-RPE cell types. Taken together, these results confirmed our in vitro findings that RPE could detect intracellular RNA via RIG-I.

### 3.3. Expression of cGAS Was Not Detectable in Retinal Pigment Epithelium

The cGAS DNA sensor has been reported to play a major role in sensing the Alu-RNA-induced IFN response and degeneration of RPE, in part through sensing mitochondrial DNA [4]. We were thus surprised that knockdown of cGAS was shown to have little effect on the DNA-induced production of IFN within RPE (Figures 2(b) and 2(c)). Upon further investigation, cGAS transcripts were undetected in iPS-RPE, which are more similar to primary RPE cells, and detected only at very low levels in ARPE-19 cells (at least 1000-fold lower than housekeeping gene controls). Our findings were supported by recent analyses of single-cell transcripts from human donors [27] showing that cGAS was not detectable in RPE and only enriched in myeloid cells (Supplementary Figure 6). Next, we evaluated the level of the cGAS protein in RPE in vitro to confirm the lack of expression of this gene. We measured the level of the cGAS protein using a cGAS antibody validated in THP1-Dual KO-cGAS cells (Supplementary Figure 7A). Accordingly, we did not detect any cGAS protein in either ARPE-19 or iPS-RPE cells (Supplementary Figure 7A). We observed a similar pattern using ELISA, where cGAS was detected in THP-1 cells but undetectable in negative control THP1-Dual KO-cGAS cells and below the limit of detection in either ARPE-19 or iPS-RPE cells (Supplementary Figure 7B).

Previous reports suggested that cGAS was part of the induced ISG response machinery following activation of type I IFN response [28, 29]. However, when nucleic acids were used to stimulate cells in vitro, we were unable to detect the expression of cGAS in either ARPE-19 or iPS-RPE cells (Supplementary Figure 7A and 7B). This suggested that cGAS might not be expressed in RPE and was not induced in RPE following exposure to nucleic acids.

It has been reported that the cGAS protein might predominantly reside in the nucleus, interacting with, and potentially being sequestered by, genomic DNA [30]. To exclude this possibility within RPE, we applied high doses of salt to dissociate proteins and genomic DNA. We used histones as a control to show that chromatin-bound proteins were released after treatment with high-dose sodium chloride; however, cGAS was still undetectable (Supplementary Figure 7C). Collectively, these results confirmed that RPE have an undetectable expression of cGAS and consequently cannot directly respond to DNA stimulation through cGAS (Figures 2(b) and 2(c)).

### 3.4. Activation of Interferon Induced Cell Death-Related Gene Expression and Altered the Barrier Function of Retinal Pigment Epithelium in Response to RNA

**Figure 6:**
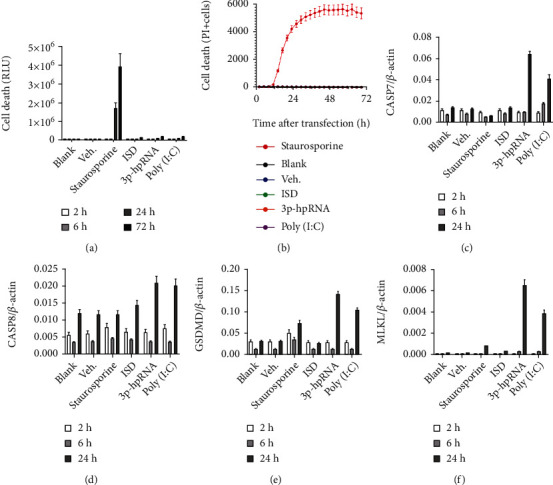
Nucleic acids do not induce RPE cell death but increase pro-cell death pathway genes. (a, b) Cell death in nucleic acid-transfected iPS-RPE cells (iCell) was measured by CytoTox-Glo (a) or propidium iodide (PI) staining (b). Staurosporine (10 *μ*M) was used as positive control to induce apoptosis. None of the tested nucleic acids (1 *μ*g/mL) induced RPE death. (c–f) Cell death pathway-relevant genes CASP7, CASP8, GSDMD, and MLKL were measured by qPCR in iCell at indicated times after transfection of nucleic acids (1 *μ*g/mL). 3p-hpRNA and poly(I:C), but not ISD, induced upregulation of pro-death genes.

In order to evaluate whether activation of the interferon pathway by nucleic acids can induce RPE cell degeneration, we measured iPS-RPE cell death using the following two methods: (1) enzyme release from dead cells by CytoTox-Glo and (2) real-time cell death monitoring by propidium iodide (PI) staining using IncuCyte. While cell death was observed in response to the positive control staurosporine, cell death was not observed in cells treated with nucleic acids (Figures 6(a) and 6(b)). The data suggest NA-mediated activation of the IFN pathway does not result in immediate RPE cell death.

We then examined the expression of cell death pathway genes, as it has been reported in other settings that type I IFN response can prime cells to become more sensitive toward cell death by upregulating the expression of key nodes in cell death pathways [31–35]. Indeed, as shown in Figures 6(c)–6(f), gene expression of key effectors of various types of cell death, including CASP7/8 (apoptosis), Gasdermin-D (GSDMD, pyroptosis), and MLKL (necroptosis), was induced upon RNA stimulation. This data supports the hypothesis that NA sensing in RPE cells may sensitize the cells to secondary insults by upregulating cell death effectors during degeneration during disease progression and eventually lead to RPE cell death.

**Figure 7:**
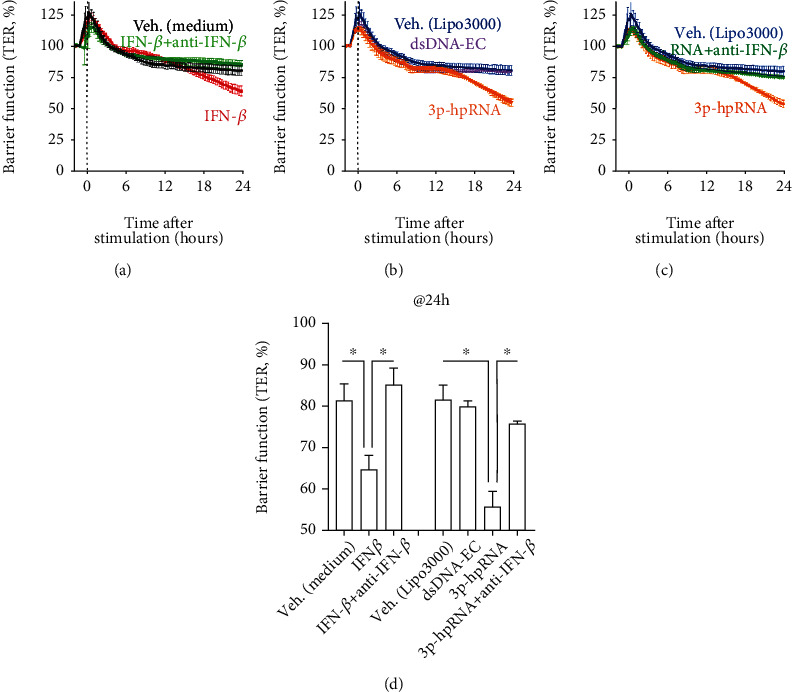
Type I IFN response alters RPE barrier function. iPS-RPE cells were cultured on transmembrane in 24-well plates, and TER was measured as an indicator of RPE barrier function. Note that treatment with IFN-*β* at 100 ng/mL (a) or transfection with RNA but not DNA at 0.25 *μ*g/mL (b) could reduce TER, whereas anti-IFN-*β* could recover the IFN-*β*-induced (a) and RNA-induced loss of barrier function (c). Normalized TER after 24 h treatment is summarized in (d) as a bar graph indicating changes to the barrier function. Each data point represents biological replicates (*n* = 3). ^∗^*p* < 0.05 compared with IFN-*β*- or 3p-hpRNA-induced group with TER loss.

Furthermore, to better understand the biological consequence of elevated type I IFN response on RPE cells, we set out to investigate the RPE barrier, as this is a key RPE cell function [36, 37]. In order to evaluate the effects of IFNs on RPE barrier function, we measured the transepithelial resistance (TER) in polarized iPS-RPE cells cultured on a permeable transmembrane matrix. As shown in Figures 7(a) and 7(d), the barrier function was impaired in cells treated with IFN-*β*, and, as expected, an anti-IFN-*β* antibody was able to prevent this IFN-*β*-induced leakage. Consistent with our findings, RNA but not DNA was shown to be capable of impairing TER (Figures 7(b) and 7(d)). We were able to further validate that the nucleic acid sensing-induced impaired barrier function was dependent on secreted IFNs, as addition of an anti-IFN-*β* antibody was sufficient to prevent the loss of TER in RPE (Figures 7(c) and 7(d)). These results further supported our findings that the IFN response in RPE cells is induced through RNA but not DNA sensors.

## 4. Discussion

Type I IFN signaling is known to play a critical role in host defense, especially in infectious diseases. Increasing evidence has suggested a role for the type I IFN response in chronic diseases with sterile inflammation, especially autoimmune diseases and aging-related diseases, such as systemic lupus erythematosus, multiple sclerosis, Sjogren's syndrome, atherosclerosis, and Alzheimer's disease [38–42]. Our data, in agreement with previous reports [4, 5], showed that this pathway is also involved in AMD, an age-related degenerative disease. In our study, RIG-I, one of the key node-sensing nucleic acids and an ISG in the IFN response, was significantly upregulated in patients with GA. In agreement with previous publications [4], our data revealed an increased type I IFN response in retina, exhibited by increased levels of IFN-*β* in RPE of patients with GA.

There are many innate receptors responsible for sensing nucleic acids and activating the type I IFN pathway. In particular for RNA sensing, TLRs, especially TLR3, have been reported to recognize dsRNA to initiate an IFN response in RPE, with the TLR3 412Phe variant showing protective effects against GA [19, 43, 44]. Human RIG-I, encoded by DDX58, is a dsRNA helicase enzyme that has been demonstrated to play important roles in RNA sensing. In this study, we showed that there was no IFN response detected in cells responding to nucleic acids without transfection reagents except for poly(I:C) (Figure 2(b)), which is consistent with previous reports showing that TLR3 is responsible for extracellular poly(I:C)-induced cytokine release in RPE [10, 11]. Here, we hypothesized that both TLR3 and RIG-I might be important for initiating intracellular poly(I:C)-induced NF-*κ*B-dependent release of cytokines from RPE cells (Figure 3(b)). However, we found that knockdown of members of the TLR signaling pathway (e.g., TLR3, MYD88 innate immune signal transduction adaptor (MYD88), and Toll-like receptor adaptor molecule 1 (TICAM1, known as TRIF)) had no effect on IFN-*β* production (Figure 2(c)), but knockdown or knockout of RIG-I abolishes IFN-*β* and IL-6 production in RPE (Figures 2(c), 3(a), and 3(b)), suggesting that RIG-I but not TLR3 is the key sensor for initiating a type I IFN response in responding to intracellular RNA in RPE.

While the production of type I IFN is known to be caused by viral and bacterial infections, it might also be the result of chronic sterile inflammation. Based on our results using bacterial dihydrodipicolinate reductase (dapB) as a negative control for RNAscope (Supplementary Figure 1D), we excluded the possibility of bacterial contamination in our human donor samples and considered that the elevation of type I IFN response observed in patients with GA was due to sterile inflammation. Endogenous nucleic acids, for example, might be abnormally released into the cytosol during cellular dysfunction and might then be recognized by corresponding sensors, resulting in the induction of different inflammatory mediators via specific signaling pathways [45, 46]. While both DNA and RNA might potentially activate IFN pathways, when studying this possibility in RPE cells, which are unique to the retina, we found that RNA, but not DNA, except for poly(dA:dT) as discussed above, induced an IFN response (Figure 2(b)). There might be several potential sources of RNA inducers in patients with AMD. For instance, mitochondrial dysfunction was highlighted as one of the main features observed in patients with AMD [47]. In addition to mtDNA release from dysregulated mitochondria triggering a type I IFN response via the cGAS-STING pathway [46], it was reported that mitochondrial double-stranded RNA (mtdsRNA) could trigger a type I IFN response through RIG-I and the melanoma differentiation-associated protein 5 (MDA5) RIG-I-like receptor [45]. Additionally, DICER dysregulation and Alu RNA accumulation were reported in patients with AMD, suggesting that this accumulation might cause mitochondrial dysfunction, mtDNA release, and subsequent initiation of the IFN response via the cGAS-STING pathway [4].

Our study, in contrast, showed that cGAS was not involved in nucleic acid sensing in RPE cells. Multiple methods were used, and we were unable to confirm any detectable expression of cGAS in either basal or induced RPE cells. Therefore, it is more likely that accumulated RNA directly activated type I IFN response in RPE cells via the RNA sensor RIG-I. This finding was in agreement with recently published results on human retina profiling using single-nucleus RNA-seq (NucSeq) [27]. This dataset was internalized and reanalyzed. Briefly, RIG-I (DDX58) is known to be widely expressed in most cells, including RPE, and even more highly enriched in astrocytes and Muller cells. However, interestingly, cGAS (MB21D1) is known to be only expressed in myeloid cells, but not RPE (Supplementary Figure 6), further supporting our findings on the lack of expression of cGAS in RPE (Supplementary Figure 7).

There might be several potential explanations for the discrepancies observed between reports on the activity of RIG-I and cGAS in the retina: (1) RPE cells might directly respond to Alu-RNA via RIG-I, potentially initiating a type I IFN response and then signaling to professional inflammatory cells, such as macrophages or microglia, to amplify this response in a cGAS-dependent manner; (2) macrophages/microglia might initiate retinal damage via the cGAS/STING pathway and then activate/prime RPE cells to be more sensitive to RIG-I-recognized RNA inducers, thus helping amplify the signal to affect the whole retina. That is, other cell types, particularly macrophage/microglia, might be activated through cGAS/STING and subsequently lead to activation and damage of RPE cells. Therefore, it would be interesting to further explore the mechanisms by which other cell types in the retina communicate and are coactivated alongside the RPE.

The elevation of IFN signatures (i.e., ISGs), which is observed in patients with GA perhaps as a consequence of nucleic acid sensing, has been shown to be a sign of both activated IRF3-mediated type I IFN response and NF-*κ*B-mediated release of cytokines through nucleic acid sensors and the TBK1 essential mediator [8, 9, 23–25]. The induction of IFN pathway genes, including cell death effectors and other ISGs, could amplify the signal through a positive feedback loop. This might include key nodes of the IFN pathway, such as cGAS, RIG-I, interferon regulatory factor 7 (IRF7), and signal transducer and activator of transcription 1 (STAT1) [26, 48, 49], which could increase the sensitivity of the sensing of nucleic acids and thus keep activating NF-*κ*B and IRF3-mediated inflammation. Here, we showed that in addition to type I IFN response, the NF-*κ*B-mediated release of cytokines was also activated in RPE cells in response to nucleic acid sensing, specifically RNA (Figure 3(b)). Additionally, the RNA-induced release of cytokines was abolished in RIG-I KO cells, indicating that the secretion of cytokines in response to nucleic acid sensing was also RIG-I-dependent in RPE.

Interestingly, we observed the upregulation of RIG-I transcripts across the entire retina and RPE-choroid, and not confined to atrophic regions in patients with GA (Figure 4(a)). As RIG-I is one of the ISGs, this data suggested that IFN-mediated inflammation was amplified in the whole retina of patients with GA. It is possible that activation of type I IFN response in RPE cells may prime the cells and lead to further cell degeneration/death across the retina by upregulating ISGs, such as mixed lineage kinase domain-like pseudokinase (MLKL), caspase 8 (CASP8), and TNF superfamily member 10 (TNFSF10, known also as TRAIL) [31–35]. In our study, we found that 3p-hpRNA and poly(I:C) could induce CASP7/8, GSDMD, and MLKL gene expression (Figures 6(c)–6(f)), further supporting that although IFN activation did not directly induce RPE death, it induces key nodes of cell death pathways and may prime RPE cells toward death. This is consistent with the slow progressive nature of RPE degeneration in AMD and GA, where it may take years and often decades to develop atrophy and cause vision loss.

In the clinic, IFN-*α* is commonly used for the treatment of patients with hepatitis C, whereas IFN-*β* is used as a first line immune-modulatory treatment for multiple sclerosis. While IFN-*α* and IFN-*β* have been reported to exhibit similar systemic side effects, retinopathy is a frequently observed adverse effect of IFN-*α*, with only a limited number of case reports on IFN-*β*-associated retinopathy being reported [50, 51]. Interestingly, the previously reported issue of ocular toxicity resulting from siRNA therapy [44] has been raised again for adeno-associated viral vector- (AAV-) based gene therapy [52]. A potential explanation, in addition to IFN-mediated effects on cell death, might be that IFN might impair RPE barrier function, as shown in our study (Figure 7).

**Figure 8:**
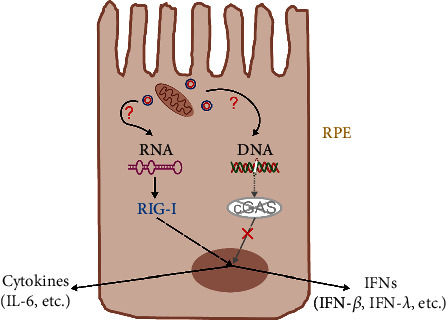
Schematic diagram for potential mechanism of intracellular nucleic acid sensing in RPE.

In summary, we identified the RIG-I/DDX58 RNA sensor as the major sensor for intracellular nucleic acids in RPE cells. A schematic diagram to illustrate a potential mechanism is summarized in Figure 8. Our results illustrated the mechanism by which type I IFN response functions in RPE cells, and especially the means by which distinct cell types might contribute to common molecular mechanisms via different signaling pathways, thus giving new clues for the improved understanding of the mechanisms of action at the single-cell level through in vivo observations. The results of this study might help explain the mechanism through which nucleic acids can induce retinal degeneration and thereby identify a potential therapeutic target to reduce the IFN response and improve or delay retinal degeneration.

## Data Availability

The authors confirm that the data supporting the findings of this study are available within the article and/or its supplementary materials.

## Abbreviations

AMD: Age-related macular degeneration
RPE: Retinal pigment epithelium
IFN: Interferon
ISG: Interferon-stimulated gene
GA: Geographic atrophy
TLR: Toll-like receptor
DAMPs: Danger-associated molecular patterns
PRRs: Pattern recognition receptors
dsDNA: Double-stranded DNA
TER: Transepithelial resistance.

## Supplementary Materials

Supplementary Materials Table S1: patient information for this study. Figure S1: method optimization and validation for IHC and RNAscope. Figure S2: iPS-derived RPE cells show RPE phenotypes. Figure S3: the method of generation and validation of an ARPE-19-Cas9 cell line. Figure S4: examples of RIG-I mRNA (by RNAscope, (a)) and protein (by IHC, (b)) expression level from a GA patient (donor 6). Different areas were selected to show in higher magnifications. Figure S5: represented pictures of RIG-I IHC from 10 donors (5 non-AMD controls and 5 GA patients) show consistent upregulation of RIG-I proteins in GA patients to support Figure 5. Figure S6: RIG-I and cGAS expression patterns in human non-AMD control retina. Figure S7: no functional cGAS is detected in RPE.
