# Supplementary material for: Mechanism of Nucleic Acid Sensing in Retinal Pigment Epithelium (RPE): RIG-I Mediates Type I Interferon Response in Human RPE
Source: J Immunol Res. 2021 Jun 18;2021:9975628. doi: 10.1155/2021/9975628 (PMC8235977; doi:10.1155/2021/9975628)
Supplement: Supplementary Materials — Table S1: patient information for this study. Figure S1: method optimization and validation for IHC and RNAscope. Figure S2: iPS-derived RPE cells show RPE phenotypes. Figure S3: the method of generation and validation of an ARPE-19-Cas9 cell line. Figure S4: examples of RIG-I mRNA (by RNAscope, (a)) and protein (by IHC, (b)) expression level from a GA patient (donor 6). Different areas were selected to show in higher magnifications. Figure S5: represented pictures of RIG-I IHC from 10 donors (5 non-AMD controls and 5 GA patients) show consistent upregulation of RIG-I proteins in GA patients to support Figure 5. Figure S6: RIG-I and cGAS expression patterns in human non-AMD control retina. Figure S7: no functional cGAS is detected in RPE. [file 9975628.f1.pdf]

Supplementary material for online publish only

Supplementary Table 1. Patient information used for IHC and RNAScope.

Patient ID	Age	Gender	Macular Pathology
donor1	67	M	normal
donor2	78	M	normal
donor3	83	F	normal
donor4	73	M	normal
donor5	84	M	normal
donor6	93	F	GA
donor7	94	F	GA
donor8	90	M	GA
donor9	85	F	GA
donor10	81	M	GA

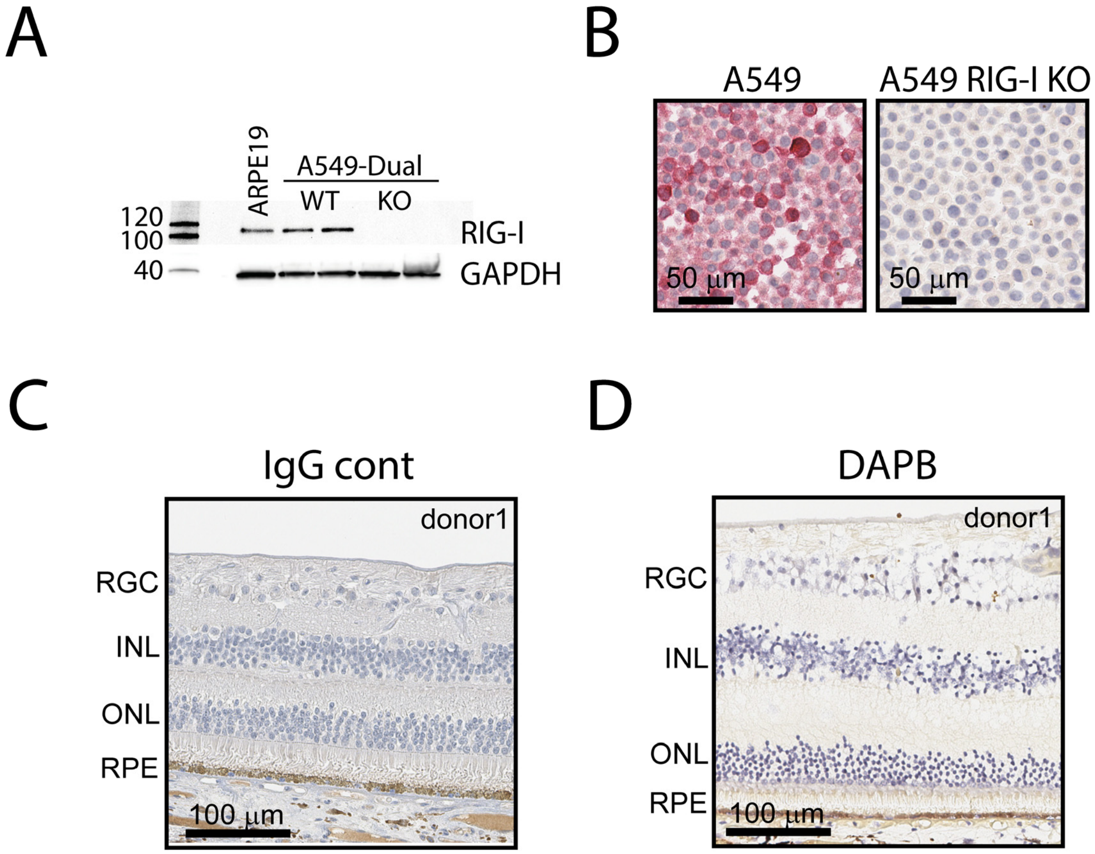

Supplementary Figure 1. Method optimization and validation for IHC and RNAscope. (A-B) The IHC antibody used for RIG-I was validated in A549-Dual RIG-I KO cells. The KO cell line was validated by western blotting (A), and then the RIG-I antibody was validated in these KO cells by IHC. (C-D) Negative control for IHC and RNAscope. The IgG control was used as negative control for IHC (C) and DAPB was used as negative control for RNAscope (D).

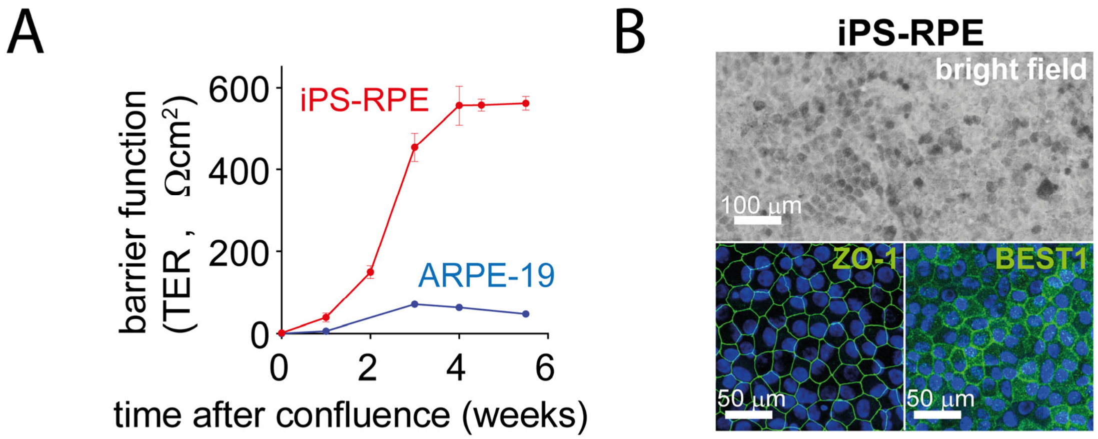

Supplementary Figure 2. iPS-derived RPE cells show RPE phenotypes. (A) The barrier function of iPS-RPE and ARPE-19 cells was measured using TER. Note that iPS-RPE shown much stronger barrier function than ARPE-19. (B) iPS-RPE cells show pigmentation, ZO-1, and BEST1 positive staining, indicating RPE phenotypes.

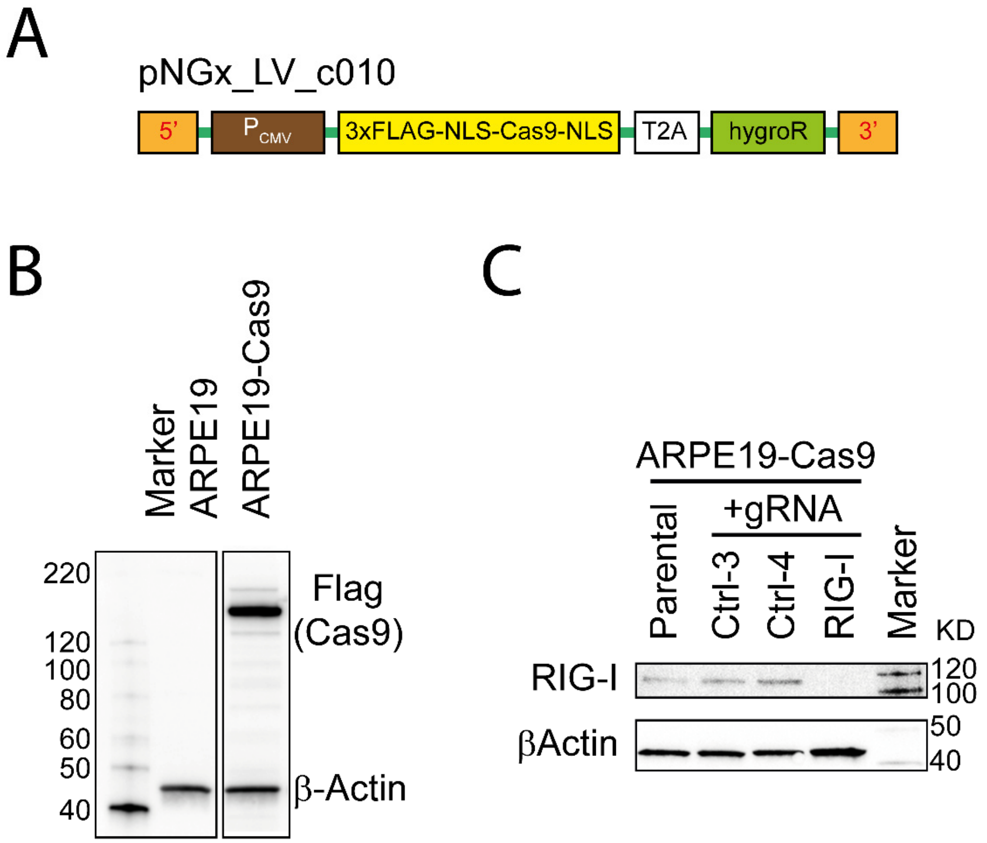

Supplementary Figure 3. Generation and validation of ARPE-19 RIG-I KO cell line. (A-B) Generation and validation of ARPE-19-Cas9 cell line. (A) Schematic of coding sequence introduced by Cas9 viral vector pNGx_LV_c010. (B) Anti-flag western blot showing expression of flag-tagged Cas9 protein in transduced ARPE-19 cells. Extraneous western blot lanes were cropped out. (C) The ARPE-19 RIG-I KO cell line was validated using western blot.

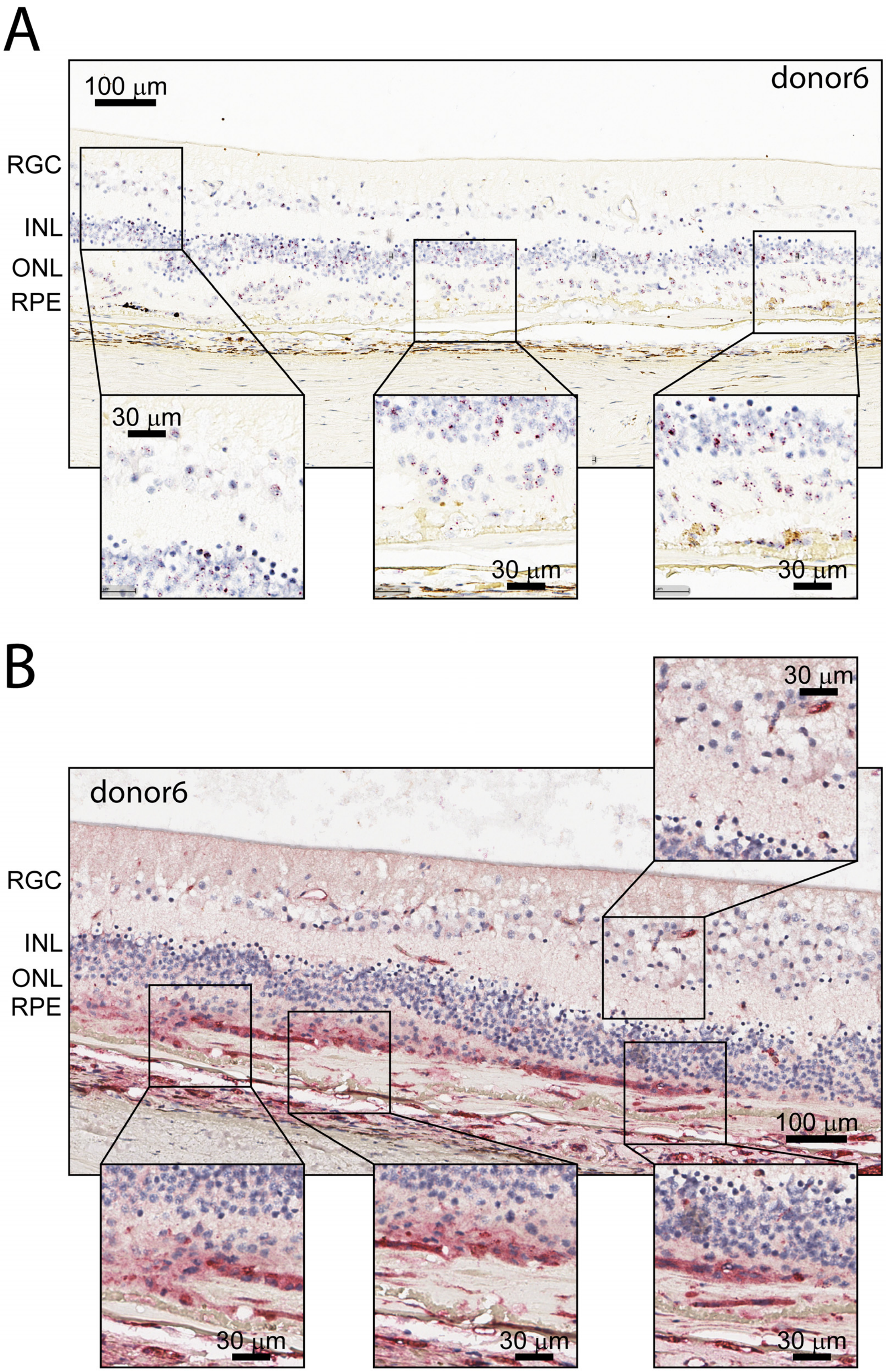

Supplementary Figure 4. Examples of RIG-I mRNA (by RNAscope, A) and protein (by IHC, B) expression level from a GA patient (donor 6). Different areas were selected to shown in higher magnifications.

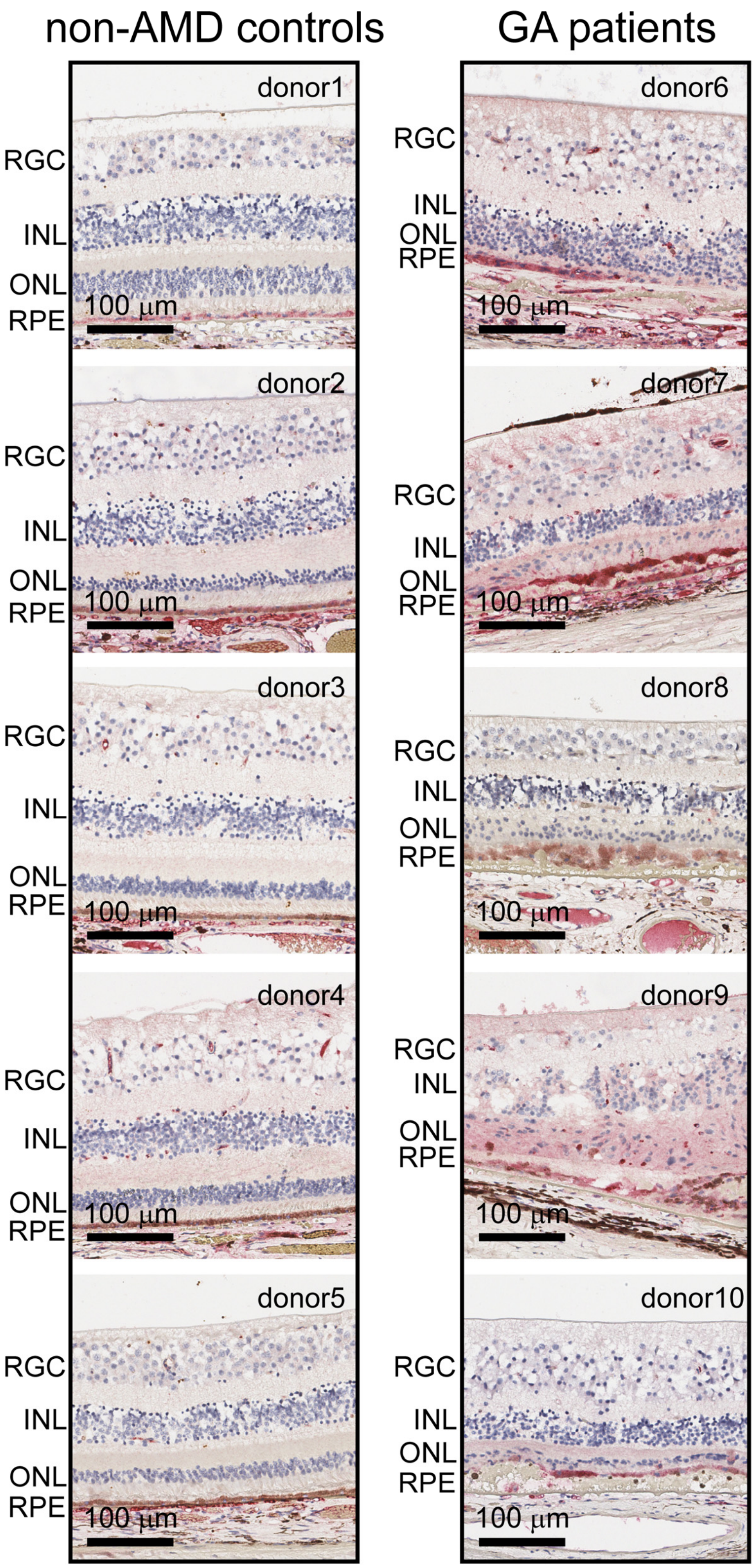

Supplementary Figure 5. Represented pictures of RIG-I IHC from 10 donors (5 non-AMD controls and 5 GA patients) shows consistent upregulation of RIG-I proteins in GA patients.

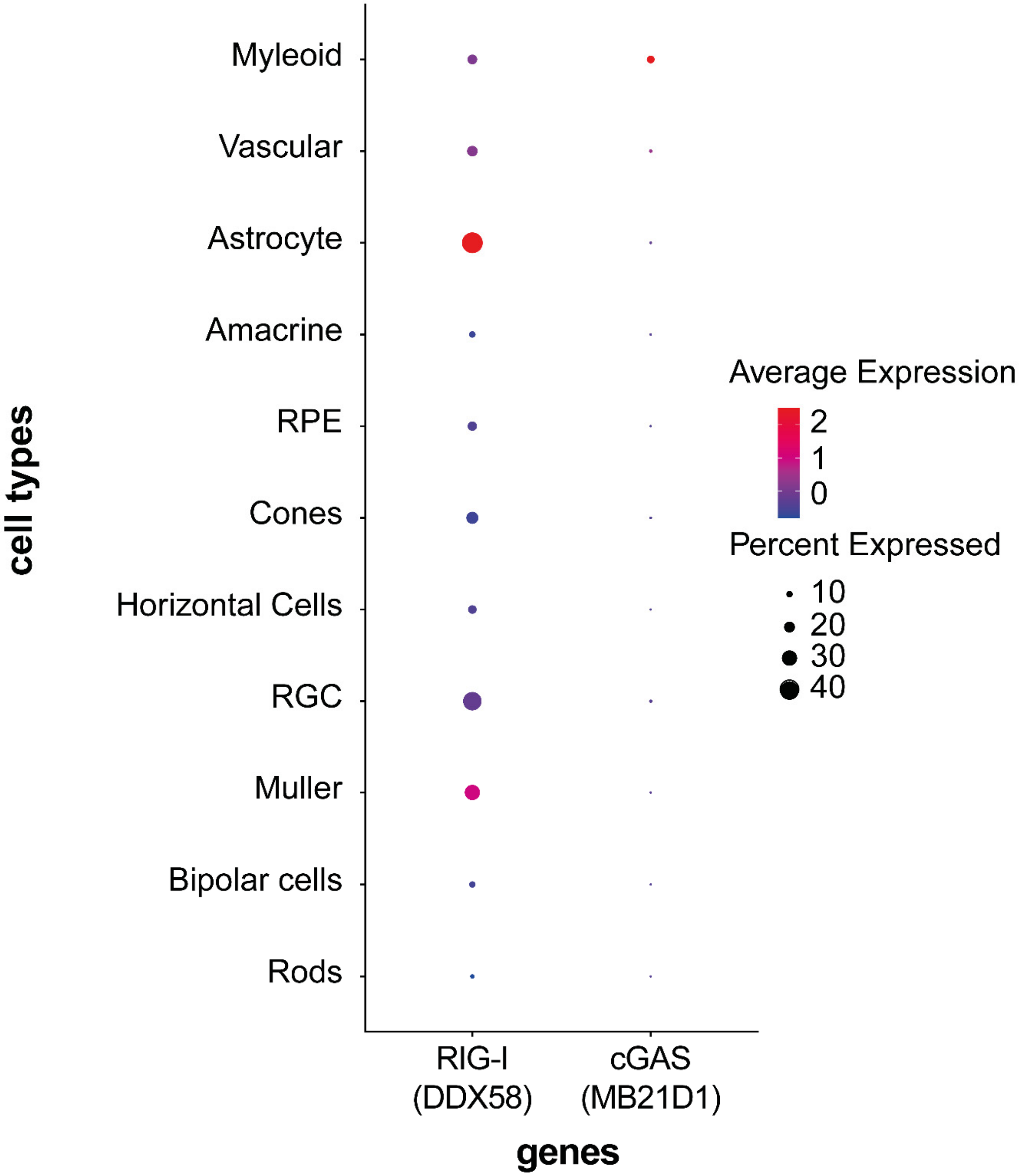

Supplementary Figure 6. RIG-I and cGAS expression pattern in human non-AMD control retina were re-analyzed from a published single-nucleus RNA-seq (NucSeq) dataset (PMID: 31995762. Processed NucSeq data can be found in GEO: GSE135133. NucSeq results can be browsed in the Broad Institute's Single-Cell Portal under study SCP484 at https://singlecell.broadinstitute.org/single_cell). Note that RIG-I (DDX58) is widely expressed in most cell types including RPE, and enriched even more highly in astrocytes and Muller cells. However, cGAS (MB21D1) is only expressed in myeloid cells, but not RPE. RGC- retinal ganglion cells; RPE- retinal pigment epithelium.

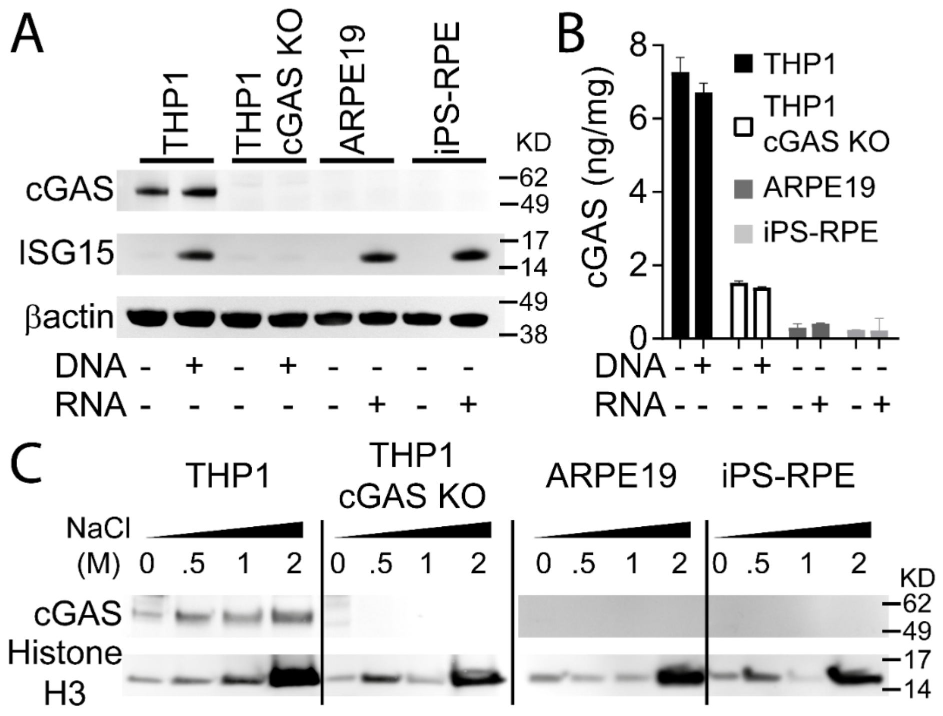

Supplementary Figure 7. No functional cGAS is detected in RPE. (A-B) No cGAS protein was detected in RPE cells using western blotting (A) and ELISA (B) under both basal and stimulated (0.25 μg/mL DNA or RNA as indicated) conditions. Note that the cGAS antibody was validated using THP-1 and THP-1-cGAS KO cells. (C) No cGAS was detected in the nuclei of RPE. Indicated concentrations of sodium chloride used to dissociate proteins from chromosomes. Histone H3 was used as positive control to indicate the release of chromatin-bound proteins in nuclear fractionation. Extraneous lanes in western blotting were cropped out.
